# Tryptophan Pathway Abnormalities in a Murine Model of Hereditary Glaucoma

**DOI:** 10.3390/ijms22031039

**Published:** 2021-01-21

**Authors:** Michal Fiedorowicz, Tomasz Choragiewicz, Waldemar A. Turski, Tomasz Kocki, Dominika Nowakowska, Kamila Wertejuk, Agnieszka Kamińska, Teresio Avitabile, Marlena Wełniak-Kaminska, Pawel Grieb, Sandrine Zweifel, Robert Rejdak, Mario Damiano Toro

**Affiliations:** 1 Mossakowski Medical Research Centre, Polish Academy of Sciences, 02-106 Warsaw, Poland; mfiedorowicz@imdik.pan.pl (M.F.); marlenak@imdik.pan.pl (M.W.-K.); pgrieb@imdik.pan.pl (P.G.); 2 Department of General Ophthalmology, Medical University of Lublin, 20-079 Lublin, Poland; tomekchor@wp.pl (T.C.); dominika.nowakowska85@gmail.com (D.N.); kamwer@gmail.com (K.W.); robertrejdak@yahoo.com (R.R.); 3 Department of Experimental and Clinical Pharmacology, Medical University of Lublin, 20-079 Lublin, Poland; turskiwa@op.pl (W.A.T.); tomek94@poczta.onet.pl (T.K.); 4 Faculty of Medical Sciences, Collegium Medicum, Cardinal Stefan Wyszyński University, 01-815 Warsaw, Poland; agnieszka.kaminska73@wp.pl; 5 Department of Ophthalmology, University of Catania, 95123 Catania, Italy; t.avitabile@unict.it; 6 Department of Ophthalmology, University Hospital Zurich, University of Zurich, 8091 Zurich, Switzerland; sandrine.zweifel@usz.ch

**Keywords:** glaucoma, kynurenine pathway, DBA/2J, retina

## Abstract

Background: It has been shown that a possible pathogenetic mechanism of neurodegeneration in the mouse model of glaucoma (DBA/2J) may be an alteration of kynurenic acid (KYNA) in the retina. This study aimed to verify the hypothesis that alterations of tryptophan (TRP) metabolism in DBA/2J mice is not limited to the retina. Methods: Samples of the retinal tissue and serum were collected from DBA/2J mice (6 and 10 months old) and control C57Bl/6 mice of the same age. The concentration of TRP, KYNA, kynurenine (KYN), and 3-hydroxykynurenine (3OH-K) was measured by HPLC. The activity of indoleamine 2,3-dioxygenase (IDO) was also determined as a KYN/TRP ratio. Results: TRP, KYNA, L-KYN, and 3OH-K concentration were significantly lower in the retinas of DBA/2J mice than in C57Bl/6 mice. 3OH-K concentration was higher in older mice in both strains. Serum TRP, L-KYN, and KYNA concentrations were lower in DBA/2J than in age-matched controls. However, serum IDO activity did not differ significantly between compared groups and strains. Conclusions: Alterations of the TRP pathway seem not to be limited to the retina in the murine model of hereditary glaucoma.

## 1. Introduction

Glaucoma is the most frequent cause of irreversible blindness worldwide [1,2,3]. It is associated with retinal ganglion cell (RGC) degeneration and loss. Moreover, neurodegeneration in glaucoma is not limited to RGCs and their axons, forming the optic nerve, but seems to affect numerous brain structures, particularly vision-related brain structures [2,3,4,5,6,7]. The mechanism of neurodegeneration in glaucoma is not sufficiently elucidated, and in particular, the mechanisms of this so-called extraretinal neurodegeneration are poorly understood [7,8].

One of the possible mechanisms involved in glaucomatous neurodegeneration is activation of the major metabolic pathway for tryptophan (TRP) metabolism, the kynurenine pathway (KP), or shift in the balance between the three KP branches metabolizing the central KP metabolite, kynurenine (KYN). Some of the KP metabolites are known to display neurotoxic (e.g., quinolinic acid, QUIN; the agonist of the NMDA receptor [9]) or neuroprotective properties (e.g., kynurenic acid, KYNA; the antagonist of the NMDA and the alpha-7 nicotinic acetylcholine receptors [10,11]). KP is also essential for energy metabolism. Additionally, it may contribute to the modulation of the immune response [12].

Retinal KYNA levels were shown to be elevated in response to RGC damage [13]. As we recently have shown, the dynamics of KP alteration due to RGC damage is different depending on the nature of the insult (or animal model of RGC loss used) [14]. Importantly, recent results suggest that manipulation in KP enzymes activity may exert neuroprotective effects on RGC [15].

It would be highly important to evaluate KP metabolites in a glaucoma model that would resemble the situation in chronic, age-related disease, like the majority of glaucoma cases. Such a model would also enable tracking systemic changes in KP metabolites levels, not only in the retina. The DBA/2J murine strain mimics pigmentary glaucoma. In this strain, glaucomatous alteration develops slowly with age and is not restricted to the retina but also affects other vision-related brain structures [16]. DBA/2J mice carry mutations in tyrosinase-related protein 1 gene (*Tyrp1^isa^*) and glycoprotein nonmetastatic melanoma protein B gene (*Gpnmb^R150X^*). These mutations induce anterior segment anomalies such as iris atrophy, pigment dispersion syndrome, and anterior synechia that result in disturbances in the aqueous humor outflow at the trabecular meshwork, IOP elevation [17,18,19,20] as well as RGCs loss and alterations of the ocular morphology [17,21]. They also develop age-related failure of the anterograde axonal transport within the RGC axons [22,23]. We have previously shown that KP is affected in the course of spontaneous retinal degeneration in this glaucoma model [24,25]. However, no data are available on the systemic changes in TRP metabolism and KP in the course of spontaneous glaucoma in these mice.

This study aimed to verify the hypothesis that alterations of TRP metabolism in DBA/2J mice are not limited to the retina but will also be detectable in the serum.

## 2. Results

### 2.1. Retina

**Figure 1:**
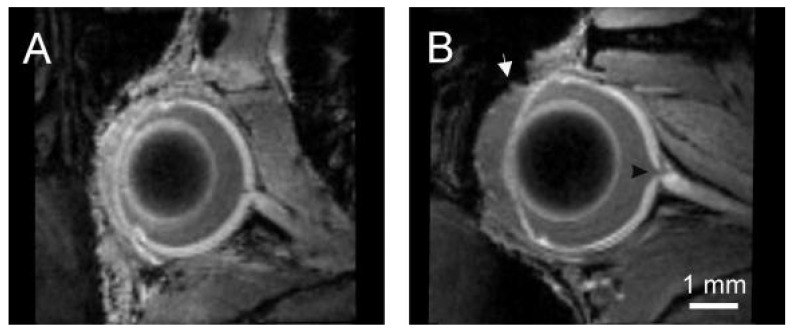
Representative T1-weighted magnetic resonance images of the eyes in 10-month-old C57BL/6 (**A**) and DBA/2J (**B**) mice 24 h after administration of a manganese contrast agent. The black arrowhead indicates the optic disc cupping (black arrowhead). White arrow—enlarged anterior chamber of the eye in DBA/2J.

In 10-month-old DBA/2J mice, magnetic resonance imaging (MRI) revealed deepening of the anterior chamber (Figure 1). Retina visualization after systemic administration of manganese chloride also showed optic disc cupping (Figure 1B, arrowhead) that was not visible in C57Bl/6 mice (Figure 1A).

#### 2.1.1. Kynurenic Acid

**Figure 2:**
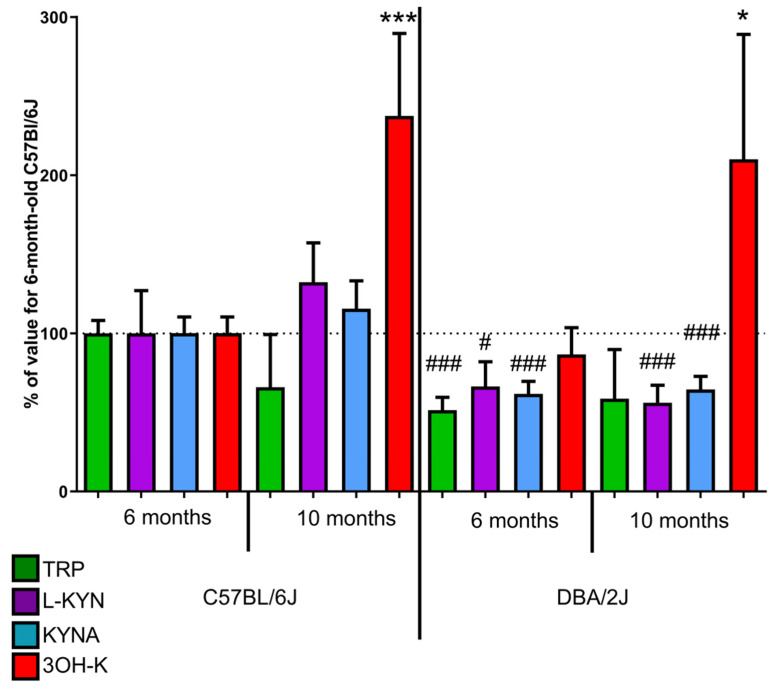
The levels of tryptophan (TRP), L-kynurenine (L-KYN), kynurenic acid (KYNA), and 3-hydroxykynurenine (3OH-K) in the retinas of C57BL/6 and DBA/2J mice. Values are expressed as a percentage of TRP, L-KYN, KYNA, or 3-OH-K concentrations in samples from 6-month-old C57Bl/6 mice. ^#^ *p* < 0.05, ^###^ *p* < 0.001 DBA2J vs. C57Bl/6; * *p* < 0.05, ***p < 0.001 10 months vs. 6 months.

Both in 6-month and 10-month-old DBA/2J mice, a significantly lower concentration of retinal KYNA was observed (12.95 and 13.55 pmol/mg) than in C57BL/6 mice. (21.01 pmo/mg, *p* < 0.001 and 24.27 pmol/mg. *p* < 0.001). No statistically significant differences were observed between 6- and 10-month-old DBA/2J mice (Figure 2).

#### 2.1.2. 3-Hydroxykynurenine

Levels of 3OH-K were higher both in 6- and 10-month-old DBA/2J mice (4.42 and 3.91 pmol/mg, respectively) than in C57BL/6 mice (1.86 pmol/mg, *p* < 0.001 and 1.61 pmol/mg, *p* < 0.05, respectively). Both in DBA/2J and C57BL/6 mice, concentrations in 6 months old animals were significantly lower than in 10 months (*p* < 0.01 and *p <* 0.05, respectively) (Figure 2).

#### 2.1.3. Tryptophan

Retinal TRP concentration observed in 6 months old DBA/2J mice (19.13 pmol/mg) was significantly lower than in C57BL/6 mice (37.23 pmol/mg, *p* < 0.001). The difference in concentration in 10-month-old mice was not statistically significant between the two groups (24.50 pmol/mg vs. 21.84 pmol/mg; Figure 2).

#### 2.1.4. L-Kynurenine

Retinal levels of L-KYN both in 6- and 10-month-old DBA/2J mice (1.54 and 1.30 pmol/kg) were significantly lower than in C57BL/6 mice (2.33 pmol/mg, *p* < 0.05 and 3.08 pmol/mg, *p* < 0.05). No statistically significant differences were observed between 6- and 10-month-old DBA/2J mice (Figure 2).

### 2.2. Serum

#### 2.2.1. Kynurenic acid

**Figure 3:**
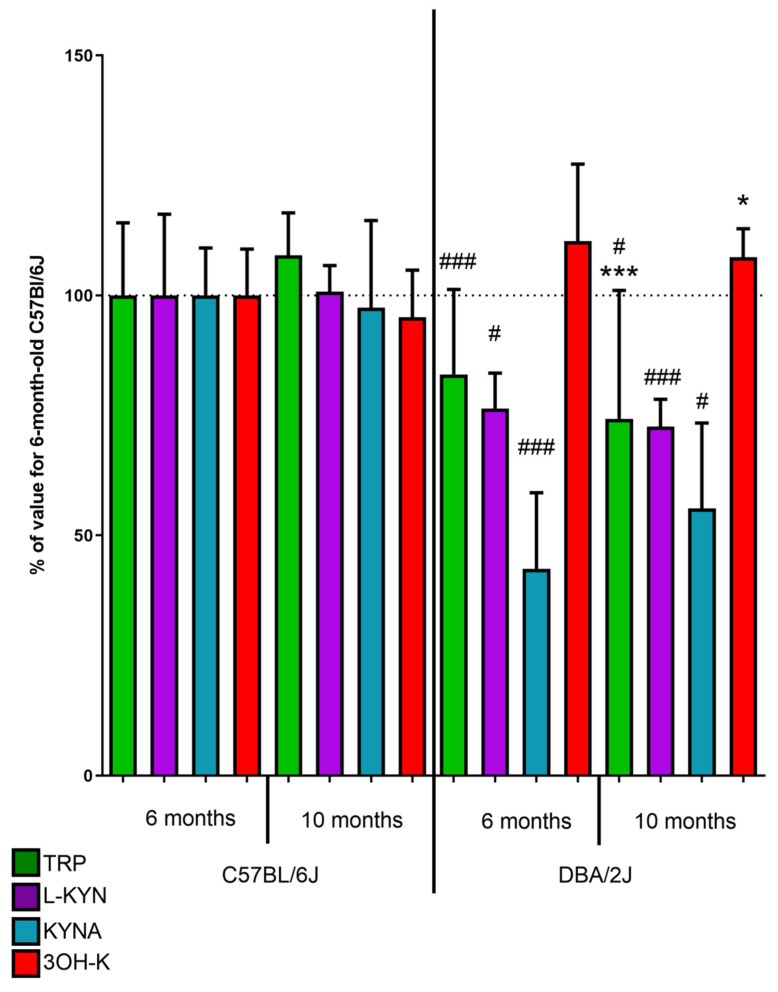
The levels of tryptophan (TRP), L-kynurenine (L-KYN), kynurenic acid (KYNA), and 3-hydroxykynurenine (3OH-K) in the serum of C57BL/6 and DBA/2J mice. Values are expressed as a percentage of TRP, L-KYN, KYNA, or 3-OH-K concentrations in samples from 6-month-old C57Bl/6 mice. ^#^ *p* < 0.05, ^###^ *p* < 0.001 DBA2J vs. C57Bl/6; * *p* < 0.05, ***p < 0.001 10 months vs. 6 months.

Both 6-month and 10-month-old DBA/2J mice had a significantly lower concentration of KYNA in serum (5.15 and 6.66 pmol/mL) when compared to C57BL/6 control mice (11.96 pmol/mL, *p* < 0.001 and 11.66 pmol/mL, *p* < 0.05). No statistically significant differences were observed between 6- and 10-month-old DBA/2J mice (Figure 3).

#### 2.2.2. 3-Hydroxykynurenine

No statistically significant difference was observed in 3OH-K serum concentration in 6-month-old DBA/2J mice (14.38 pmol/mL) compared to 6-month-old C57BL/6 mice (12.91 pmol/mL). In contrast, in 10-month-old DBA/2J mice, serum concentration was significantly higher (13.95 pmol/mL) than in C57BL/6 mice of the same age (12.33 pmol/mL, *p* < 0.05). A significantly lower 3OH-K concentration was observed in 10-month-old DBA/2J mice than in 6-month-old animals of this strain (*p* < 0.05, Figure 3).

#### 2.2.3. Tryptophan

In both 6- and 10-month-old DBA/2J mice serum level of TRP (673.13 and 598.89 pmol/mL) was lower than in C57BL/6 mice (805.76 pmol/mL, *p* < 0.001 and 872.74 pmol/mL, *p* < 0.05). Concentration in the serum of 10-month-old DBA/2J mice was significantly lower (*p* < 0.01) than in 6-month-old mice (Figure 3).

#### 2.2.4. L-Kynurenine

Both 6-month and 10-month-old DBA/2J mice had a significantly lower concentration of L-KYN in serum (138.09 and 139.23 pmol/mL) when compared to C57BL/6 control mice (105.49 pmol/mL, *p* < 0.001 and 100.34 pmol/mL, *p* < 0.05).

### 2.3. IDO activity

**Figure 4:**
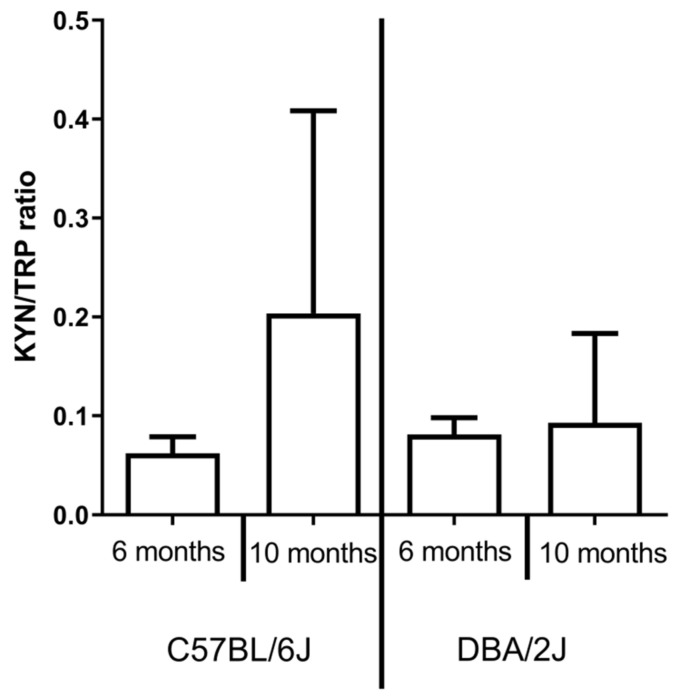
Indoleamine 2,3 dioxygenase (IDO) activity was expressed as a kynurenine/tryptophan ratio in the retina of C57BL/6 and DBA/2J mice.

**Figure 5:**
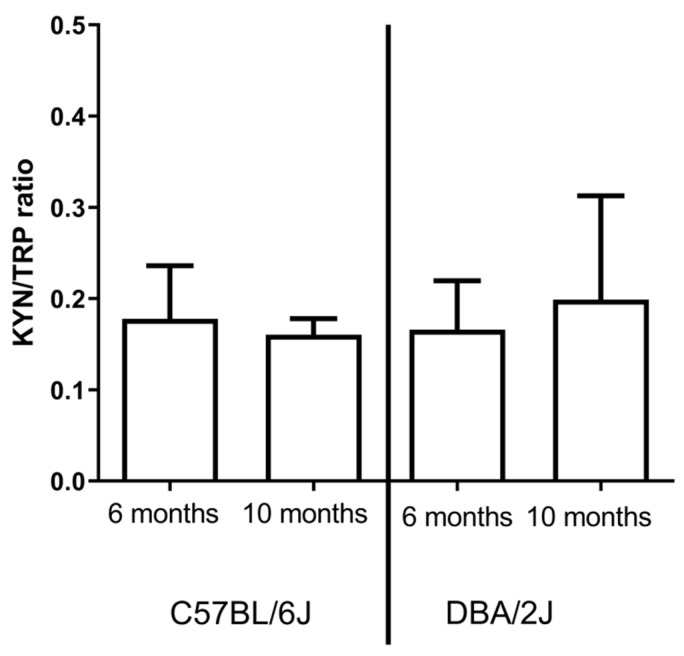
Indoleamine 2,3 dioxygenase (IDO) activity was expressed as a kynurenine/tryptophan ratio in the serum of C57BL/6 and DBA/2J mice.

No significant differences in IDO activity were observed between 6- and 10-month-old DBA/2J and C57BL/6 mice both in the retina (Figure 4) and serum (Figure 5).

## 3. Discussion

DBA/2J mice are widely used as a glaucoma model, presenting secondary angle-closure glaucoma caused by pigment debris deposition and blockage of the aqueous fluid outflow that develops spontaneously in an age-dependent manner [24]. In these mice, we evaluated KP metabolites concentrations, both in the retina and in the serum. We showed that the pattern of differences between the control mice and glaucoma model as well age-dependent differences were generally similar in the retina and serum. Interestingly, no age-dependent 3OH-K elevation was observed in the serum in contrast to the retinas.

DBA/2J mice develop several age-related pathologies. IOP elevation in these mice is observed around the 6th month of age, and after the 12th month of age, IOP starts to decrease. In addition, alterations in ocular morphology like the elongation of the eyeball, deepening of the anterior chamber, or increased iridocorneal angle begin around the 6th month [26,27,28,29,30,31]. At the 10th month, these alterations were already clearly visible in the present study. In this study, we also noticed signs of retinal neurodegeneration in 10-month-old mice, i.e., optic disc cupping.

Disease progression in the DBA/2J glaucoma model is associated with RGC degeneration and loss at the later pathology stages. Apoptotic RGCs were found as early as in 3-month-old animals [21]. However, RGC density was shown to remain relatively stable up to 12 months of age [32], despite a dramatic decrease in visual acuity demonstrated in 11-month-old animals [33]. Therefore, it seems that RGC degeneration rather than loss and axonal transport failure contribute to the pathological changes in the retina and the optic nerve in these mice at moderately advanced stages of the disease [22].

Some data indicate that pathological processes start earlier than noticeable IOP elevation, even as early as in 3-month-old animals [22,34]. Nevertheless, DBA/2J performance in visual tasks seems similar to C57BL/6 mice at 6 months and significantly decreased when the mice are older [33,35]. Therefore, we selected two timepoints that seem representative for the pathology in this glaucoma model – mice at the early stage of disease (6-month-old) and with moderately advanced glaucoma (10-month-old). As controls for the murine model of glaucoma, we used C57BL/6J mice. This strain is frequently used as a control for DBA/2J mice in experimental ophthalmology studies [16,22,27,35]. C57BL/6 mice develop only mild age-related IOP elevation and do not normally display detectable abnormalities in the ocular morphology [22,27,36]. We did not observe any abnormalities reported to sporadically develop in these mice [37] like microphthalmia/anophthalmia or corneal opacity.

Alterations in KP may contribute to retinal degeneration in DBA/2J mice. The levels of KYNA, the neuroprotective KP metabolite, were lower in the retinas of DBA/2J mice than in control retinas; that result is in accordance with our previous results [24]. However, in this study, we did not detect an age-related decrease in KYNA concentration in these mice. It may be an effect of slightly earlier timepoints proposed in the current study. Our previous results also demonstrated changes in retinal KYNA in two surgical models of glaucoma. A complex response was noted after NMDA administration (a transient increase 2 days after NMDA injection and then a decrease) and a massive decrease of retinal KYNA concentration due to partial optic nerve crush [14]. The decrease in KYNA concentration observed in this study seems to be an effect of KP failure. Not surprisingly, we did not observe a transient increase in KYNA levels that could reflect endogenous anti-excitotoxic defense mechanisms. We believe that the present experimental setup, a slowly progressing model of hereditary glaucoma, might be close to the situation in clinics.

A lower level KYNA, as well as TRP and L-KYN, might result from decreased levels of these metabolites in the serum that we noted in this study. However, KYNA was thought to cross the blood-retina barrier (BRB) hardly [38]; in DBA/2J mice, intravenous administration of KYNA resulted in RGC neuroprotection, which can suggest BRB permeability in DBA/2J mice due to decoupling of gap junctions [39] and reduction of pericytes [40]. In our experiment, the calculated IDO activity did not differ from that in controls both in the retina and in serum. However, this might be an effect of the measurement method or the selected timepoints. For example, elevated IDO expression has been shown in the retina in the course of diabetic retinopathy due to microglial activation [41]. Therefore, the elevation of IDO activity might be present at some pathology stages in DBA/2J mice.

In contrast to other KP metabolites, levels of 3OH-K, a KP metabolite that is thought to play a neurotoxic role in several pathologies [42], were elevated in the retina in 10-month-old DBA/2J mice when compared to 6-month-old mice. Remarkably, we did not observe a similar elevation of this metabolite in the serum. Conversion of KYN to 3OH-K is catalyzed by kynurenine 3-monooxygenase (KMO), an enzyme produced in the nervous system in microglia [43]. Microglia activation was previously shown to be an early event in the course of glaucomatous pathology in DBA/2J mice and to correlate with the severity of neurodegeneration in these mice [44]. We have also recently shown that KMO is overexpressed in two animal models of glaucoma: optic nerve crush and NMDA-induced RGC loss [14]. Interestingly, the dynamics of changes differed in these two animal models (peak expression 12 h after crush and 48 h after intravitreal NMDA administration). 3OH-K concentration, therefore, may correspond with pathological degenerative changes observed in DBA/2J mice. In the 6th month, apoptosis peak was noticed, followed by necrosis, Mueller glia cell activation, and angiogenesis [21]. These observations suggest that KMO overexpression and activation could be a common mechanism in glaucomatous neurodegeneration. Studying this mechanism is an interesting new direction since the data on the role of KMO activation and overproduction of 3OH-K in retinal pathologies are currently very scarce.

Besides ocular abnormalities, DBA/2J mice also display numerous other pathologies such as hearing loss [45], susceptibility to audiogenic seizures [46], and calcified lesions [47]. At least some of these abnormalities may be related to TRP metabolism disturbances; an example could be audiogenic seizures that were shown to be linked with abnormal serotonin signaling in these mice [46]. Considering the number of pathologies and reduced concentration of the KP metabolites in the serum, DBA/2J mice seem to be a good candidate model for further experiments investigating these correlations.

Finally, modulating the TRP metabolism was postulated as a new therapeutic target in multiple neurological diseases [48]. One promising approach is the development of KP enzymes inhibitors. While IDO inhibitors have already reached clinical trials, they are rather regarded as potential anti-cancer therapeutics; KMO inhibitors that could be of more interest as potential drugs in neurodegenerative diseases are still in the earlier pre-clinical stage of development [49]. However, the data on the therapeutic modulation of KP in retinal degeneration is still limited. A study by Harper et al. demonstrated that administration of a KMO inhibitor Ro-61-8048 prevented retinal injury induced by supersonic blast waves [50]. Another recent study by Nahomi et al. [15] demonstrated that the absence of KMO is protective against RGCs in retinal ischemia-reperfusion injury (induced by transient elevation of IOP). Moreover, they demonstrated the protective effect of intravenous KYNA administration despite some reports suggesting low permeability of the brain-blood barrier and possibly also blood-retina barrier to KYNA [38]. Our results indicate that such an approach could also be promising in animal models of glaucoma and, finally, as a therapeutic target for future clinical applications. Moreover, DBA/2J mice could be an attractive animal model for studying such interventions’ therapeutic efficacy.

One limitation of the study is the relatively small number of animals included. However, it was possible to demonstrate statistically significant differences between the groups. We have also decided to include only female mice, which allowed us to make the study groups homogenous and reduce the number of animals required according to the 3R rule. However, we cannot exclude the effect of sex on TRP metabolism. It would also be interesting to track TRP metabolism alterations in older animals with very advanced glaucoma. In the future, it would also be desirable to perform a direct measurement of IDO activity that would allow a more definite conclusion on changes of IDO activity in the DBA/2J glaucoma model.

The content of TRP recorded in our blood samples was low in comparison to data from literature [51,52,53]. The reason for this discrepancy is unknown. It is well known that quantification of TRP is a real challenge and different methods have been developed and utilized. Thus, the use of different methods of sample preparation and tryptophan detection might explain at least in part the differences between data reported by different groups of researchers. However, we are convinced, that apart from the absolute values, the comparison of data obtained under identical experimental conditions are conclusive.

Finally, it was not possible to perform a histological evaluation of the retinas that underwent biochemical analysis. Therefore, we cannot directly confirm RGC degeneration in all the DBA/2J mice included in the study. However, our previous results showed alterations in ocular morphology and elevated IOP in virtually all 10-month-old animals from the same population [27].

## 4. Materials and Methods

### 4.1. Animals

All experiments were performed according to the ARVO Statement for the Use of Animals in Ophthalmic and Vision Research and the European Communities Council directive of 22 September 2010 (2010/63/EU). All the respective local regulations for animal experiments were also obeyed, and the respective local ethics committee approved the experiments (Warsaw IV Local Ethics Committee, approvals no. 88/2012, 94/2012, 11/2015).

The experiment was performed in female DBA/2J mice aged 6 and 10 months (model of glaucoma, 5 animals per group) and female C57BL/6 mice of the same age (control mice, 5 animals per group) that were obtained from the breeding colony at the Mossakowski Medical Research Centre Polish Academy of Sciences (MMRC). John et al. [26] have shown a significant impact of sex on IOP in DBA/2J mice. In this study, the difference was significant from the 6th month of age (13 mm Hg in females vs. 9.4 mm Hg in males) and was even more pronounced in 9-month-old animals (20.3 mm Hg vs. 16.2 mm Hg). Therefore, we chose to perform this experiment on females only as in our previous studies [22,27] to make the groups more consistent in terms of IOP-related pathology.

Animals were provided a standardized diet and water ad libitum and were maintained at 12–12 h light-dark cycle.

### 4.2. Sample Collection

Animals were anesthetized with ketamine-xylazine injection (ip., 100 mg/kg body weight and 10 mg/kg, respectively). Blood was collected from the right ventricle of the heart after thoracotomy. Afterward, both eyeballs were extracted, and the death of the animal was confirmed by cervical dislocation. Eyes underwent hemisection along the ora serrata. The cornea, lens, and vitreous body were subsequently removed, and the retinas were collected and immediately frozen in liquid nitrogen. Blood samples (0.7–1.0 mL) were allowed to clot for 2 h and afterward centrifuged (10 min, 1100× *g*, 4 °C). The retina and serum samples were stored at −80 °C until the determinations of KP metabolites were performed.

### 4.3. Determination of Tryptophan and its Metabolites

One hundred microliters of blood samples were deproteinized with twenty microliters of 6% M HClO4 and centrifuged at 12,000× *g* for 20 min at 4 °C.

TRP, KYN, and KYNA were measured with high-performance liquid chromatography (HPLC) according to Zhao et al. (2010) with modifications [54]. The UltiMate 3000 analytical HPLC system with UltiMate 3000 Autosampler with column compartment (Thermo Fisher Scientific, Waltham, MA, United States) was used for the measurements. Chromatography was carried out at 30 °C. The HPLC system was equipped with an analytical column (Agilent HC-C18(2) column; 250 × 4.6 mm i.d.; 5 µm particle size, i.d. volume per injection was 100 μL) was controlled by Chromeleon software. The mobile phase used for the separation of the samples (20 mmol/L NaAc, 3 mmol/L ZnAc2, and 7% acetonitrile) was pumped at a flow-rate of 1 mL/min; (the volume per injection was 100 µL). KYN and TRP were detected with a UV detector (set for 365 nm and 250 nm, respectively). KYNA was analyzed fluorometrically (excitation 344 nm, emission 398 nm).

3OH-K was analyzed using an electrochemical detector (The Thermo Scientific Dionex UltiMate 3000 ECD-3000RS), connected to an analytical cell with the oxidation voltage set at 0.6 V, according to the method described by Heyes and Quearry [55]. Waters Spherisorb S3 ODS2 150 × 2.1 mm column (USA) was perfused with a mobile phase consisting of 2% acetonitrile, 0.9% triethylamine, 0.59% phosphoric acid, 0.27 mM sodium EDTA, and 8.9 mM heptane sulfonic acid (flow 0.3 mL/min; the volume per injection was 10 µL). Chromeleon 7.2 software was used to control HPLC systems and record the chromatographic data. IDO activity was calculated as a KYN/TRP ratio.

The stock solutions of TRP and kynurenines were prepared by dissolving the relevant amount of working standards in the mobile phase. The concentration of the standard was equal to or greater than the maximum concentration to be found in samples. All stock solutions were stored at 2–8 °C. The formula used for calculating the calibration curve was the external standard method. The six-point linearity curve was prepared in three replicates. Thermo Scientific Chromeleon Chromatography Data System automatically determined the calibration curves for the external standards. For TRP and kynurenines quantification, the reference calibration curves were used. TRP and kynurenines were identified based on their retention times (TRP 12.2 ± 0.3 min; L-KYN 6.4 ± 0.3 min; KYNA 11.2 ± 0.2 min; and 3OH-K 6.8 ± 0.4 min).

The concentrations were calculated by comparing the peak area of the sample with the same area of the standard used commercially. In order to evaluate the HPLC method, every 1 out of 10 samples was chosen at random and spiked with known concentrations of mixed standards in duplicate. For the calculation of the recovery percentage, we used the following equation: % recovery = 100 × (measured value for spiked samples − measured value for unspiked samples)/known value of the mixed standards. The results include samples which recovery percentage is in the range from 80% to 120%.

### 4.4. Visualization of the Neural Retina and Ocular Morphology with Magnetic Resonance Imaging

The neural retina was visualized, as we described previously [27]. In brief, the animals received an intraperitoneal injection of 0.1 mol/L manganese chloride solution and were subjected to magnetic resonance imaging (MRI) after 24 h. Imaging was performed with 7T MR tomograph (BioSpec 70/30USR Bruker, Ettlingen, Germany) equipped with 86 mm (inner diameter) transmit coil and 10 mm planar receive-only surface coil. The imaging sequence used was T1-weighted FLASH 3D (TR/TE = 48/4 ms, NA = 5, FA = 25°, spatial resolution 68 μm × 68 μm × 68 μm, scan time 28min). This imaging protocol also allowed us to visualize ocular morphology in these mice.

### 4.5. Statistical Analysis

Shapiro–Wilk test was applied for normality, followed by a nonparametric Mann–Whitney test or parametric Student’s *t*-test by a normal distribution. Differences were considered statistically significant for *p* < 0.05. Data are presented as mean ± standard deviation.

## 5. Conclusions

In conclusion, according to our results, the TRP metabolism alterations in the retina of DBA/2J mice seem to reflect systemic alterations in TRP metabolism. Moreover, DBA/2J mice could be an attractive model for studying TRP metabolism alterations’ effects.

## Data Availability

The raw data supporting the conclusions of this article will be made available by the authors, without undue reservation.
